# Endometriosis: An Inflammatory Disease That Requires New Therapeutic Options

**DOI:** 10.3390/ijms23031518

**Published:** 2022-01-28

**Authors:** Jacques Donnez, Luciana Cacciottola

**Affiliations:** 1 Société de Recherche pour l’Infertilité (SRI), 143 Avenue Grandchamp, 1150 Brussels, Belgium; jacques.donnez@gmail.com; 2 Université Catholique de Louvain, 1200 Brussels, Belgium; 3 Gynecology Research Unit, Institut de Recherche Expérimentale et Clinique, Université Catholique de Louvain, Avenue Mounier 52, 1200 Brussels, Belgium

Endometriosis, defined as the presence of endometrium-like tissue outside the uterus, is estimated to affect 10% of women of reproductive age. The 2008 US healthcare costs for endometriosis were approximately USD 4000 per affected woman, which are similar to those of other chronic diseases such as type 2 diabetes, rheumatoid arthritis, and Crohn’s disease. There are three distinct forms of the disease (peritoneal, ovarian, and recto-vaginal endometriosis), each of them being associated with specific symptoms, although dysmenorrhea and chronic non-menstrual pelvic pain are the most prevalent [1]. The postulated origins of endometriotic lesions are retrograde menstruation, celomatic metaplasia, and lymphatic and vascular metaplasia [2]. Identifying the exact pathogenesis of endometriosis is challenging and contentious for gynecologists and reproduction specialists, endocrinologists, and researchers. There is a huge need for comprehensive understanding to detect and investigate the events that take place in the microenvironment of the uterus in order to characterize the various pathogenetic mechanisms, which contribute to the causes and development of endometriosis.

The goal of this Special Issue was to evaluate some new biological targets as well as new therapeutical approaches.

In their original research, Gamisonia et al. [3] investigated the effect of endocannabinoid-like compounds from the N-acyl dopamine (NADA) family on the viability of stromal cells forming ectopic and eutopic endometrium of patients with ovarian endometriosis. Their in vitro study demonstrated a dose-dependent cytotoxic effect of NADAs on stromal cells of eutopic and ectopic endometrium. The effects of all investigated NADAs were pronounced on cell viability after 5 h of incubation. The authors suggest that cell death mechanisms provoked by NADAs may be the NADA-induced generation of reactive oxygen species (ROS), as they were able to block NADA toxicity by using antioxidants. There are some limitations, as explained by the authors themselves: (i) the ectopic endometrial cells originated from ovarian endometrioma and it cannot be stated that a similar cytotoxic effect of NADAs can be observed for endometriotic stromal cells from other localizations; (ii) the activity of NADAs observed in vitro should be confirmed and reproduced in vivo. Complementary research is therefore needed to assess this therapeutic approach.

In an experimental rat model of endometriosis, Siracusa et al. [4] aimed to assess the effects of autophagy and mitophagy, namely the selective autophagy of abnormal and/or damaged mitochondria, in the progression and development of endometriosis. Indeed, it has been described that autophagy activation could inhibit the progression of endometriotic lesions. It has been demonstrated in several in vivo experiments that the inhibition of autophagy downregulated apoptosis. The reported results [4] showed that rapamycin administration, by activation of the autophagy and mitophagy pathways, leads to the (i) activation of apoptosis and (ii) inhibition of the mechanistic target of rapamycin (mTOR) with a subsequent decrease in the proliferation of endothelial cells (marked by vascular endothelia growth factor (VEGF) and CD34). However, their research has limitations. The endometriosis model that was applied (transplantation of uterine tissue into the abdominal cavity) does not represent the pathogenesis of human endometriosis. Indeed, rats do not have spontaneous lesions of endometriosis and do not menstruate. The rat model is thus far away from the reality, and future experiments on human endometriotic lesions are needed to validate their findings. 

The pathologist’s perspective was reported by Camboni and Marbaix [5]. They described the numerous pathological classification systems, but unfortunately, up to the present time, none of them has been widely accepted or implemented. For the authors, there is evidence that endometriosis may have many different appearances which may, moreover, change overtime. Even if histological diagnosis of endometriosis is relatively simple, the relationship between histopathological features and the severity of the disease and symptoms is not clear. Camboni and Marbaix [5] described with accuracy that endometriosis could be classified as a potential precancerous lesion, according to the World Health Organization (WHO). The first case of malignant transformation of endometriosis to ovarian carcinoma was reported by Sampson in 1925. Strict criteria to identify such transformation, which remains infrequent (less than 1% of cases, 75% of which arising from ovarian endometriomas), were established. Although the exact cellular pathways are unknown, transformation of endometriosis towards malignancy is likely multifactorial. Adenomyosis and endometriosis is more and more often reported as associated diseases. Similarity in the hormone receptor levels, histopathology, growth factors, and MRI results are arguments in favor of this association.

In their manuscript, Kapoor et al. [2] reviewed the biological key mechanisms responsible for inducing endometriosis and examine how their ‘cross talk’ favors disease development. Inflammation is one of the mechanisms that triggers endometriosis where cell proliferation and infiltration are implicated. Macrophages are pivotal in endometriosis, and their activation with increased secretion of cytokines is crucial in the progress of lesions, proliferation, and angiogenesis. Proinflammatory cytokines (such as interleukin 1, 8, 33, nuclear factor kappa B (NF-κB) and tumor necrosis factor alpha (TNFα)) have been extensively reported in several steps of endometriosis progression. 

In their review, the role of estrogen and progesterone and their corresponding receptors is also highlighted and the concept of progesterone resistance in endometriotic lesions explains the poor efficacy of progestins and oral contraceptives in 33% of patients. The role of apoptotic, autophagic, and tumor-promoting genes/proteins is crucial in the survival of endometriotic cells, and their pathways may represent promising targets for the development of novel therapeutic options. E-cadherin, N-cadherin, and B-catenin are regulators of the epithelial–mesenchymal transition (EMT), leading to the development of endometriosis in primates and humans. Pharmacological inhibitors targeting EMT could, in theory, be beneficial for endometriosis therapy. Moreover, angiogenesis ensures a proper blood supply to endometrial lesions, and VEGF is found at high levels in endometriotic lesions and peritoneal fluid from endometriosis patients, playing a role at this level.

Of course, lowering the estrogen levels may well prove effective, especially in women who failed to respond to progestins. Donnez and Dolmans [6] evaluated in their review the effectiveness of a new class of drugs, the gonadotropin-releasing hormone (GnRH) antagonists in the management of premenopausal women with endometriosis-associated pelvic pain. There is a need for long-term oral treatments capable of managing symptoms while taking into account both the main symptoms (pain and/or infertility) and the lesion phenotypes. Indeed, due to progesterone resistance, 33% of women suffering from endometriosis are poor responders to oral contraceptives and progestogens, and their percentage climbs even higher in women with deep nodular endometriosis. Donnez and Dolmans reviewed the most important papers reporting the results from clinical trials on three potentially useful oral GnRH antagonists: elagolix, linzagolix, and relugolix [6]. These studies confirmed that GnRH antagonists suppress ovarian function in a dose-dependent manner, allowing the modulation of estrogen levels which, according to the threshold hypothesis, may provide relief from endometriosis-associated symptoms while reducing side effects such as hot flushes and bone mineral density loss. As stressed in their paper, the main advantages of GnRH antagonists, when compared to GnRH agonists, are: (i) oral administration, (ii) immediate suppression of FSH and LH secretion, (iii) dose-dependent estrogen suppression, and (iv) rapid reversibility and recovery of hormone secretion after discontinuing treatment.

In their review, Brichant et al. [7] described the multiple types of non-coding RNAs (ncRNAs) and particularly the micro RNAs (miRNAs), which are short ncRNAs of about 20–24 nucleotides. The ncRNAs display different regulatory functions, interfering into a larger RNA communication network that finally controls fundamental effectors proteins of cellular functions. Up- and down-regulation of miRNA expression in endometriotic tissue correlates with a dysregulated expression of several target proteins relevant to the pathogenesis of endometriosis. As clearly described in their paper [7], several miRNAs are involved in the process of endometriotic cell pathogenesis, endometriotic cell migration, progesterone resistance, and inflammation. Modulating miRNAs should be investigated as a future therapeutic strategy for endometriosis. Long ncRNAs are able to regulate the gene expression of various mechanisms, one of which is acting as a molecular sponge for miRNAs. Circular RNAs are highly stable compounds with regulatory function on other ncRNAs. Brichant et al. [7] conclude that non-coding RNAs are emerging key factors in the development of human diseases and play a role in endometriosis. Further studies are needed to evaluate their exact role as specific targets in medical therapy. 

In their review, Giacomini et al. [8] reported the inflammatory nature of endometriosis and the important role played by immune cells. Strong support for a causal role of inflammatory pathways in endometriosis establishment comes from the investigation of genetic factors linked to the disease. In their paper, the authors focus on the identification of the immune inflammatory targets and novel therapeutic approaches. Since the early 1980s, studies have reported the genetic component of endometriosis, when an increased prevalence of endometriosis were observed in first-degree relatives of subjects with endometriosis. Recently, genome-wide association studies (GWAS) have shed light on the genetic variants involved in the susceptibility of endometriosis. Among them, two main cellular pathways appear to be crucial in the development of the disease. The first one is the MAPK pathway, which has been suggested to favor endometriosis induction and progression via several mechanisms of (i) apoptosis, angiogenesis affecting cell growth, (ii) migration and invasion, (iii) production of inflammatory molecules and ROS, and (iv) progesterone resistance. Possible novel therapeutic approaches could be developed by the regulation of this pathway, and specifically by its inhibition, as suggested by the authors [8]. The other extensively described pathway is the WNT pathway, which plays an important role in organ homeostasis and in endometriosis. Several mechanisms were suggested to affect the disease: (i) migration and invasion, (ii) neovascularization, and (iii) production of inflammatory cytokines. Considering the WNT pathway as therapeutic target, several options could be considered, such as B-catenin degradation, the inhibition of WNT ligand binding, and transcriptional activity inhibition. 

As extensively reported by Cacciottola et al. [9], oxidative stress, namely disequilibrium between the production and neutralization of ROS, plays a key role in endometriosis pathogenesis. Indeed, excessive release of ROS induces cellular damage and likely alters cellular function by regulating gene expression and protein activity. In this review, the authors stress the pivotal role of iron, a highly toxic product resulting for lysis of erythrocytes. Activated macrophages recruited in the pelvic cavity play a vital role in the degradation of erythrocytes by phagocytosis. Senescent erythrocyte lysis is counteracted by defense mechanisms such as haptoglobin (Hp)–hemoglobin (Hb) complex for a correct iron scavenging. Nevertheless, continuous delivery of iron to macrophages may overwhelm the capacity of ferritine to store iron, causing oxidative damage to cells. Increased ROS levels in endometriosis are not only a consequence of chronic inflammation but are also caused by ROS detoxification pathway dysregulation. Indeed, a pro-oxidant environment contributes to endometriosis progression by favoring a proliferative phenotype in endometriotic lesions. A number of pathways, including the PI3K/Akt/mTOR and the Raf/MEK/ERK pathways, have been found to sustain increased proliferation of both endometrial and stroma cells in ectopic lesions. Drugs antagonizing these pathways have been shown to provoke a decline in endometriotic lesions in vitro and in experimental animal models. Among them, some drugs, such as N-acetylcysteine and resveratrol, are known for their powerful antioxidant potential. Another strategy to antagonize endometriotic lesion progression may be in using iron chelators such as desferrioxamine and erastin. Regarding endometriosis-related pain, one of the promising strategies so far was decreasing macrophage and mast cell activity. Several drugs decreasing the infiltration and activation of these immune cells have been used in experimental models, but further investigations are needed to establish whether targeting macrophages is a valid therapeutical option for endometriosis treatment. Regarding infertility, there is growing evidence that oxidative stress is involved in accelerated follicle depletion, poor oocyte quality, and lower fertility rates. Nevertheless, strategies to target ROS imbalance and modulate chronic inflammation have failed to provide a clear benefit in terms of pregnancy chances. Endometriosis-related infertility indeed remains a complex issue to address.

Recent studies, reviewed by Jiang et al. [10], have demonstrated both the ability of endometriosis to induce microbiota changes and the ability of antibiotics to treat endometriosis. The human microbiota includes all the microorganisms living in and on the body. Healthy microbiota regulates factors involved in maintaining a normal peritoneal environment and ectopic cell clearance. Dysbiosis, defined as imbalance or impairment of the microbiota which can be due to a combination of increased pathogenic microorganism and/or loss of probiotics, contributes to the dysregulation of factors responsible for endometriosis onset and progression. The gut flora is one of the richest and most studied microbiotas; it plays a crucial role in maintaining physiological gastroenteric function and is a key regulator in many inflammatory conditions.

In the uterine cavity, far less characterized microbiota exists, and its composition differs from the vaginal one and contains up to 191 operational taxonomic units. Endometrial inflammation is a major factor in the establishment and progression of endometriosis, and Jiang et al. [10] suspect that altered microbiota may be related to the disease progression. There are several postulated mechanisms of microbiota involvement in endometriosis, explained in the review: (i) bacterial contamination of the uterine environment triggers the altered inflammatory reaction observed in endometriosis, (ii) endometriosis induces gut dysbiosis by impairing gut function through cytokine activity, (iii) regulation of estrogens could be affected by gut dysbiosis, and (iv) microbiota regulates progenitor and stem cell homeostasis. The main question is whether the modulation of the microbiota may be a therapeutic approach for endometriosis. The use of antibiotics and/or probiotics is not without side effects, and such treatment to restore an ‘abnormal uterine microbiota’ remains a huge hurdle to overcome.

More than 20 years ago, we reported that peritoneal endometriosis is a chronic inflammatory disease characterized by increased numbers of peritoneal macrophages and their target products [11]. Iron overload in macrophages causes oxidative stress, which is a powerful activator of a number of proinflammatory cytokines responsible for chronic inflammation onset [12]. In this Special Issue, more and more evidence supports the role of the immune system, especially through activated macrophage function resulting in chronic inflammation and oxidative stress [4,8,9], and the need to target different inflammatory and hormonal pathways in order to reduce the establishment and progression of the disease.

## Data Availability

Not applicable.
